# Predator or provider? How wild animals respond to mixed messages from humans

**DOI:** 10.1098/rsos.211742

**Published:** 2022-03-16

**Authors:** Madeleine Goumas, Neeltje J. Boogert, Laura A. Kelley, Thomas Holding

**Affiliations:** ^1^ Centre for Ecology and Conservation, University of Exeter, Penryn Campus, Treliever Road, Penryn TR10 9FE, United Kingdom; ^2^ Max Planck Institute for Evolutionary Anthropology, Deutscher Platz 6, 04103 Leipzig, Germany

**Keywords:** human–wildlife interactions, learning rate, generalization, individual recognition, social learning, optimal foraging

## Abstract

Wild animals encounter humans on a regular basis, but humans vary widely in their behaviour: whereas many people ignore wild animals, some people present a threat, while others encourage animals' presence through feeding. Humans thus send mixed messages to which animals must respond appropriately to be successful. Some species appear to circumvent this problem by discriminating among and/or socially learning about humans, but it is not clear whether such learning strategies are actually beneficial in most cases. Using an individual-based model, we consider how learning rate, individual recognition (IR) of humans, and social learning (SL) affect wild animals' ability to reach an optimal avoidance strategy when foraging in areas frequented by humans. We show that ‘true’ IR of humans could be costly. We also find that a fast learning rate, while useful when human populations are homogeneous or highly dangerous, can cause unwarranted avoidance in other scenarios if animals generalize. SL reduces this problem by allowing conspecifics to observe benign interactions with humans. SL and a fast learning rate also improve the viability of IR. These results provide an insight into how wild animals may be affected by, and how they may cope with, contrasting human behaviour.

## 1. Introduction

Humans present a threat to a wide range of animal species, and many populations of wild animals have decreased as a result of human activity [1]. The magnitude of human impacts on biodiversity loss is predicted to worsen as the human population continues to grow [2]. While indirect effects such as anthropogenic climate change and pollution present key challenges, competition for space and overexploitation of species also have large impacts [3], revealing the importance of direct encounters between humans and wildlife. Consumptive activities, where wild animals are removed from populations through, for example, hunting or culling, are major causes of mortality. It is common for targeted animals, such as deer, to be wary of humans as a result [4]. Avoidance of humans is likely to be adaptive and protective; in areas where species are naive to humans, animals tend to be tamer and more vulnerable to human predation [5].

Although humans are a major predator of many wild animal species, numerous people coexist alongside other species without attempting to harm them. In stark contrast with other predators, humans vary widely in their behaviour towards other animals. In situations where humans do not present a threat, it is not optimal for animals to avoid humans, as doing so may result in lost foraging opportunities [6], or increased exposure to other predators [7]. When humans are not harmful, animals frequently show signs of habituation to human encounters, foraging in areas of human activity without fleeing [8]. Habituation to humans appears to be more common in urban areas where human traffic is high and predation by humans tends to be low; increased tolerance to humans has been shown in urban populations of birds, mammals and lizards [9].

Besides simply ignoring wild animals, many humans provide them with nourishment, both purposefully through targeted feeding and unintentionally by facilitating accessibility to sources of anthropogenic food (e.g. through growing crops or by disposing of waste inadequately). Intentional feeding of wild animals is often discouraged as it changes natural behaviour patterns, but is popular in many parts of the world [10]. In the United Kingdom, garden bird feeding is so prevalent that it has selected for longer bills in great tits (*Parus major*) [11]. Humans also feed animals in direct encounters [10]. As food is a necessary resource and will often be time-consuming for animals to procure, there is little doubt that direct feeding provides an incentive for animals to approach humans more closely. This potentially creates a new challenge for animals: will the next human they encounter reward them with food, ignore them or try to harm them? Some species, such as garden birds, may not be a target of consumptive activities and will be at little risk of harm. However, others, such as bears (*Ursus* spp.) and red foxes (*Vulpes vulpes*) [12], are often targets of lethal control as well as recipients of food provisioning and therefore receive what can be considered to be ‘mixed messages' from human populations.

Responding appropriately to humans is a challenge that has the potential to greatly affect the survival prospects of wild animals, yet relatively little research has been conducted on how wild animals may be able to succeed in environments where humans differ in their behaviour [13]. The relative number of humans in the population who feed versus seek to harm animals is likely to be an important determinant of how wild animals fare in human-dominated environments. Furthermore, wild animals' capacity to learn affects their subsequent behaviour and may therefore improve their ability to respond optimally to human presence [14]. As individual humans are likely to be consistent in their behaviour, discriminating among individuals rather than responding to all humans in the same way may be beneficial. There is evidence that some species can recognize individual humans. For example, northern mockingbirds (*Mimus polyglottos*) become more inclined to mob people who have disturbed their nests [15], while feral pigeons (*Columba livia*) preferentially approach people who provide food rewards [16]. Information about dangerous individual humans has also been shown to spread through wild animal populations via social learning (SL), such that animals need not experience a direct encounter with a human to respond appropriately in the future [17]. Whether such abilities to learn about humans actually benefit animals across a range of scenarios, is, however, unclear.

Here, we present an individual-based model of human–animal interactions where animals can either avoid encountering a human, or stay on their foraging ground and be subject to the human's actions, whether dangerous, rewarding or neutral. We use this model to assess the capacity for animals with different learning abilities to reach the optimal avoidance strategy within their lifetimes. We do not model the evolutionary processes that lead to these different learning strategies. Instead, we focus on how animals with already-evolved learning abilities would respond to different human populations, as behavioural plasticity is a key factor in successful adaptation to anthropogenic environments [18,19]. We first consider the factors determining the theoretical optimal avoidance strategy when animals receive mixed messages from the human population. We then ask how animals' learning rate can affect their ability to make optimal foraging decisions when encountering humans from populations that differ in their level of threat. Next, we consider how a varying ability to recognize individual humans could affect energetic outcomes. Finally, we extend the model to explore how the capacity to socially learn from conspecifics modifies avoidance behaviour.

## 2. The model

The agents in our individual-based model are referred to here as ‘critters’; these are intended to represent a generic vertebrate species that may come into contact with humans. At each discrete time step in the model, each critter encounters a randomly selected ‘human’, an agent representing a member of a human population. Each individual critter is associated with an energy value, which changes as a result of their encounters with humans. Critters begin with an energy value of 0 and can gain or lose energy as the simulation progresses. During an encounter with a human, the critter must decide either to remain on their foraging ground, i.e. ‘stay’, or to flee and ‘avoid’ the human. Each human is assigned one of the following characteristics: neutral, dangerous or rewarding. These characteristics remain constant through time and can be considered to represent a ‘type’ of human. Encounters with humans can cause a change in critter energy, simulating the effects humans have on animals in the wild. Energy should be interpreted as the result of human influence; it does not consider energy that critters would gain from foraging elsewhere. Neutral humans therefore cause no change in energy as they have no direct effect on an animal's food intake, dangerous humans cause a decrease in energy and rewarding humans cause an increase in energy. Avoiding humans causes a decrease in energy, simulating lost foraging opportunities as a result of fleeing. Each of the parameters governing change in energy for different types of encounter can be adjusted independently. There is no death in the model; therefore critters remain throughout simulation runs. Unless otherwise stated, models are run with a population of 100 humans and 500 critters and were run to equilibrium. Table 1 provides a description of parameters and baseline values used in the model. All models were run in R v. 4.0.5 [20].

**Table 1.** Baseline parameters used in the individual-based model of ‘critter’ encounters with neutral, dangerous and rewarding humans.

parameter	representation	baseline values
*N*_critters_	number of critters	500
*N*_humans_	number of humans	100
*P*_D_	proportion of the human population that is dangerous	variable
*P*_R_	proportion of the human population that is rewarding	variable
*E*_D_	energy change associated with encountering a dangerous human	−1
*E*_R_	energy change associated with encountering a rewarding human	1
*P*_A_	probability of avoiding an encounter with a human	usually initialized at 0.5; varies over time
*W*	learning weight	0.1 (low) or 0.9 (high)
*D*	discrimination (IR)	0 (no discrimination; full generalization)
		1 (full discrimination; no generalization)
*S*	SL	0 (no social learning)
		1 (full social learning)

### 2.1. Stay or avoid? Finding the theoretical optimal strategy

Wild animals must navigate a trade-off between the risk and reward associated with each possible response to encountering humans. Our simplified model considers a choice between staying and avoiding an encounter with a human. We make the assumption that the optimal strategy is one that maximizes total energy gain. The relative pay-off for staying versus avoiding is determined by the proportions of different types of human in the population and the loss or gain in energy associated with each human type. The theoretical optimal strategy can therefore be calculated by comparing the expected pay-off from staying (${\bar{E}}_{S}$) and avoiding (${\bar{E}}_{A}$) using equations (2.1) and (2.2), respectively.

2.1 $${\bar{E}}_{S}={\;P}_{D}\;\cdot \;{\;E}_{D}+{\;P}_{R}\;\cdot \;{\;E}_{R}$$

and

2.2 $${\bar{E}}_{A}=E_{A}$$

*P*_D_ is the proportion of dangerous humans in the population, *E*_D_ is the energy lost at each encounter with a dangerous human, *P*_R_ is the proportion of rewarding humans in the population, *E*_R_ represents the energy gained from encountering each rewarding human and *E*_A_ is the energy lost from avoiding an encounter.

**Figure 1.**
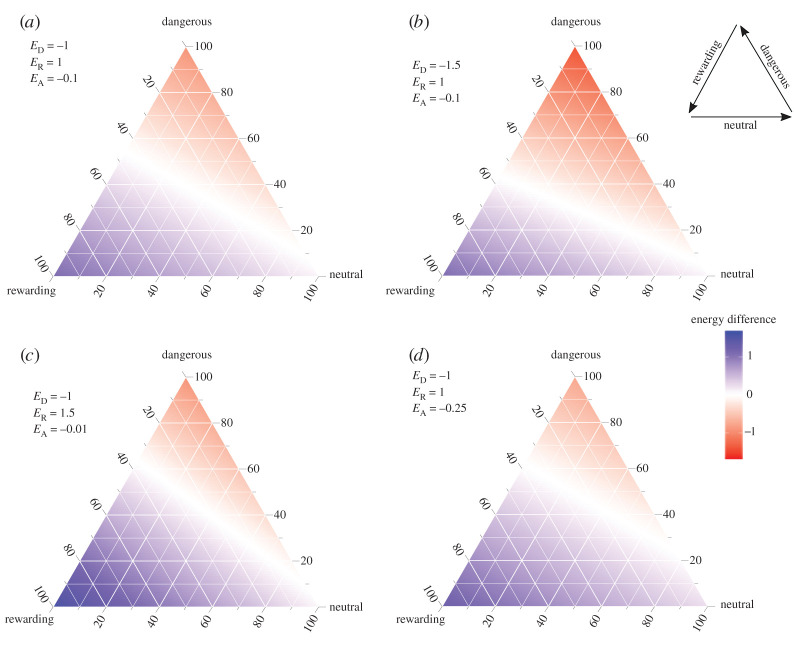
Theoretical optimal strategies to avoid or stay in scenarios of varying danger and reward. Each ternary plot shows the complete parameter space of all possible human population compositions, with proportions (as percentages) of neutral, dangerous and rewarding humans on each axis. For each set of proportions, the energy lost from avoiding an encounter with a human has been subtracted from the mean energy gained or lost by staying. Parameter space shaded red indicates scenarios where the optimal strategy is to avoid interacting with humans, parameter space shaded blue indicates scenarios where the optimal strategy is to stay and white indicates neither strategy is better than the other; (*a*) depicts a scenario where the cost of encountering a dangerous human is equal in magnitude to the benefit of encountering a rewarding human (here, a change in energy of −1 versus +1, respectively); (*b*) shows the effect of increasing the cost of encountering a dangerous human by 50% (a change in energy of −1.5); (*c*) shows the effect of increasing the benefit of encountering a rewarding human by 50% (to 1.5); (*d*) shows the effect of increasing the cost of avoiding an encounter from 0.1 (shown in (*a–c*)) to 0.25. Inset in top right shows the direction of each axis.

Figure 1 shows the theoretical optimal strategy (stay or avoid) over the complete parameter space for any given human population composition, with neutral, dangerous and rewarding humans each represented by an axis on the ternary plot. The theoretical optimal strategy is calculated by subtracting the mean energy change when avoiding from the mean energy change from staying, i.e. $\bar{E}$_S_ – $\bar{E}$_A_ . Therefore, values above zero (blue) indicate regions of parameter space where it is better to stay, values below zero (red) indicate it is better to avoid and values of zero (white) indicate that neither strategy is better than the other. Note that, since critters initially have no ability to distinguish between types of human, the theoretical optimal strategy is constrained to either ‘always avoid’ or ‘always stay’.

Our optimal strategy is determined by whether staying or avoiding results in the least amount of energy loss. The higher the proportion of dangerous humans in the population, the more critters benefit from avoiding, particularly when there are few rewarding humans to offset the costs of staying. Increasing the cost of encountering dangerous humans (i.e. figure 1*b* versus figure 1*a*) necessitates that fewer dangerous humans are needed for the cost of staying to exceed the cost of avoiding and therefore increases the range of parameter space where it is optimal to avoid humans. Similarly, increasing both the energy gained from encountering rewarding humans (figure 1*c* versus figure 1*a*) and the cost of avoiding (figure 1*d* versus figure 1*a*) increases the amount of parameter space where energy is maximized by staying. The optimal strategies derived from equations (2.1) and (2.2) provide a baseline from which we can assess how critters in the model perform in response to mixed messages from humans. In the following sections, we explore how different types of learning about humans affect critters' ability to reach an optimal avoidance strategy when encountering different people. For guidance on how to read our ternary plots [21], please see electronic supplementary material, figure S1.

## 3. Learning

As animals are able to learn to avoid aversive stimuli, approach appetitive stimuli and become habituated to harmless stimuli, we incorporated these types of learning into our model. In this section, we describe the effect of learning on the ability of critters to reach the theoretical optimal strategy. Each critter's behaviour is modelled by their probability of avoiding, which is set to 0.5 (i.e. an equal probability of staying or avoiding when encountering a human) at the beginning of the simulation. We used the following equation to update each critter's probability of avoiding a human at each encounter:

3.1 $$P_{A_{t+1}}=P_{A_{t}}\cdot (1-W)+LW,$$

where *P*_A_*t*__ is the probability of avoiding an encountered human at time *t*, *W* is the learning weight and *L* is the direction of learning. With each encounter, critter *P*_A_ is updated according to *W* and is generalized to all humans in the population*. W* controls the proportion of the updated *P*_A_ at *t* + 1 that is determined by new information from the most recent encounter and, correspondingly, how much information from prior encounters is retained. *W* therefore controls the rate of learning: a high *W* results in rapid changes in P_A_, whereas a low *W* results in smaller changes owing to more weight being placed on the accumulated information from previous encounters. Accordingly, a *W* of 0 results in no learning. For simplicity, we kept *W* uniform for all three human types, but see electronic supplementary material, figure S2 for an assessment of changing *W* independently; the interpretation of our results is not affected. *L* takes the value of 0 or 1, depending on whether the encounter encourages critters to stay or avoid, respectively. It remains constant throughout simulation runs. Encountering neutral and rewarding humans results in a tendency to stay, whereas encountering dangerous humans results in a tendency to avoid. When critters avoid a human, they are unable to learn from the encounter and their *P*_A_ at the next encounter remains the same as for the previous encounter. Note that, as long as the starting value of *P*_A_ is less than 1, it does not affect the *P*_A_ at equilibrium (see electronic supplementary material, figure S3).

### 3.1. Learning in an environment of mixed messages

When the human population is homogeneous, with all humans acting in the same manner, reaching the optimal avoidance strategy is straightforward and learning is always expected to be beneficial. In such a scenario, increasing the learning weight enables critters to reach the optimal strategy more quickly (see electronic supplementary material, figure S4 for an example). However, human populations are likely to be heterogeneous and therefore learning may not necessarily result in the best outcome. For example, when the human population is composed of both dangerous and rewarding humans, it will be difficult to converge on the optimal strategy because critters will continually receive mixed messages. We therefore considered how different human population compositions affect critter learning, energy change and, ultimately, how closely critters can approximate the optimal strategy.

**Figure 2.**
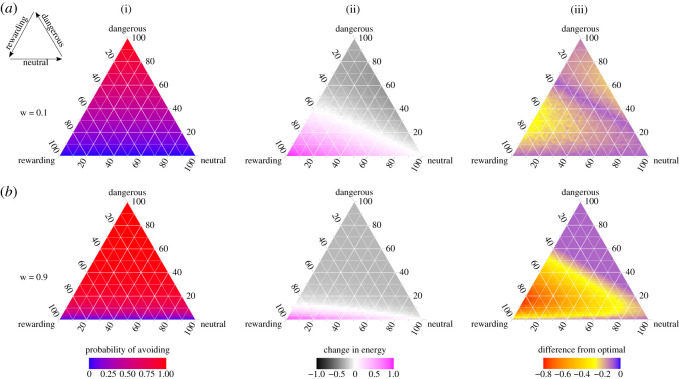
Results of model simulations where critters are able to learn to avoid dangerous humans and to stay when they encounter neutral and/or rewarding humans. Models have been run to equilibrium (200 time steps). Each ternary plot shows the complete parameter space of all possible human population compositions, with percentages of neutral, dangerous and rewarding humans on each axis. The cost of encountering a dangerous human is set to be equal in magnitude to the benefit of encountering a rewarding human (i.e. change in energy of −1 versus +1). The cost of avoiding is 0.25. Each row displays a different learning weight. Row (*a*) shows a low learning weight of 0.1 whereas row (*b*) shows a high learning weight of 0.9. Columns show different metrics for each scenario. Column (i) shows the population mean probability of avoiding an encounter with a human: blue indicates that critters tend to stay whereas red indicates that critters tend to avoid. Column (ii) shows the mean energy gained at each encounter. Parameter space shaded magenta indicates a net gain in energy and parameter space shaded grey indicates a net loss in energy, with saturation indicating the degree of loss/gain. Column (iii) shows closeness to the theoretical optimal avoidance strategy: the mean energy change at each encounter subtracted from the maximum theoretical energy possible. Blue indicates convergence on the optimal strategy and shades moving towards red show increasing distance from the optimal strategy. Inset in top left shows the direction of axes.

We first ran simulations with a relatively low learning weight of 0.1 (figure 2*a*). In this scenario, the energetic benefit of encountering a rewarding human is set to be equal in magnitude to the cost of encountering a dangerous human (i.e. the absolute value of *E*_D_ is equal to *E*_R_), and the cost of avoiding is set at 25% of the cost of encountering dangerous humans. Where the proportion of dangerous humans is intermediate between 0 and 1, critters tend to avoid at rates matching the frequency of encountering them (figure 2*a*, column i, reddish region). Only where there are relatively high numbers of rewarding humans can critters gain energy (figure 2*a*, ii, magenta region). Where there are few dangerous humans or where all humans are dangerous (i.e. there is low heterogeneity in the human population), critters converge on the optimal strategy. By contrast, when the human population is even moderately heterogeneous, critters tend to avoid when they should stay and stay when they should avoid, resulting in greater losses in energy than could otherwise be achieved (figure 2*a*, iii, yellowish regions). There is also a narrow band of blue parameter space where critters behave optimally simply because neither strategy is better than the other (see figure 1*d*). As it could be expected that animals would not learn as much from neutral encounters as they would from rewarding ones, we repeated this analysis with a lower neutral learning weight, and our results are qualitatively similar (electronic supplementary material, figure S5a).

### 3.2. Fast learning has divergent effects

It may be expected that faster learning should be beneficial. However, learning is contingent on critters staying to experience an encounter, and there is an inherent asymmetry in learning: once animals learn to avoid a stimulus, they are no longer able to gain new information about it [22]. To investigate the effect of faster learning, we repeated the simulations of figure 2*a* but with a high learning weight of 0.9 (figure 2*b*). Now, critters quickly reach a high *P*_A_ after encountering dangerous humans (figure 2*b*, column i), making it less likely that they will encounter rewarding humans in the future. Only where there are few dangerous humans do critters continue to stay for encounters, while others forfeit further learning opportunities. Consequently, a lower proportion of critters gain energy from their encounters, despite the energy parameters remaining constant (figure 2*b*, ii versus figure 2*a*, ii). Because of this learning asymmetry, it is easy for critters to reach the optimal strategy when the optimal strategy is to avoid, namely where there is a high proportion of dangerous humans (the large blue region in figure 2*b*, iii). However, fast learning also leads to a greater amount of avoidance, and greater loss of energy, when it would be beneficial to stay (the red/orange shading where rewarding humans outnumber dangerous humans; figure 2*b*, iii). Reducing the neutral learning weight results in a highly similar level of asymmetry (electronic supplementary material, figure S5b), and it is clear that it is the dangerous learning weight driving this pattern (electronic supplementary material, figure S2).

## 4. Individual recognition

Our simulations show that it is likely to be difficult for animals to maximize their foraging success in the presence of humans if they are only able to generalize from their encounters. However, some wild animal species can discriminate among individual humans and respond differently to different people in subsequent encounters, indicating that they are capable of individual recognition (IR) of humans [15,23,24]. We thus extended the model to explore the effect that IR has on critters' ability to reach an optimal avoidance strategy. We added a new parameter, *D*, which controls the degree to which critters discriminate among individual humans. When *D* > 0, critters can have a different *P*_A_ for each human. The following equations were used to update each critter's *P*_A_ for each human:

4.1 $$\Delta P_{A}=P_{A_{h,t+1}}-P_{A_{h,t}}.$$

If *h* is the individual human encountered,

4.2 $$P_{A_{h,t+1}}=P_{A_{h,t}}+\Delta P_{A}.$$

If *h* is not the individual human encountered (all other humans in the population),

4.3 $$P_{A_{h,t+1}}=P_{A_{h,t}}+(1-D)\Delta P_{A}.$$

Δ*P*_A_ is the change in *P*_A_ between the current and previous encounter (see equation (3.1) for the calculation for updating *P*_A_), and *D* is the degree to which critters discriminate among humans rather than generalize their experiences. *D* is set on a continuous scale and can be adjusted. When *D* = 0, critters completely generalize from each encounter, and *P*_A_ is updated in an identical manner for all humans, as in equation (3.1). When *D* = 1, critters fully discriminate among humans and therefore retain different, and independent, *P*_A_ values for each human. As an example of the function of the parameter, if *D* = 0.5, the *P*_A_ for an encountered human is updated identically to when *D* = 1, but half of the value of Δ*P*_A_ is also added to the *P*_A_ of all the other humans in the population, simulating some degree of generalization. This is intended to represent an animal's ability to attend to a human's unique features while also responding less strongly to features shared by all humans. For simplicity, we assume that critters retain their memory of encounters with humans throughout simulation runs.

### 4.1. The effect of individual recognition

The optimal strategy for animals with IR is to avoid only dangerous humans. Animals with perfect IR can respond appropriately to previously encountered humans as they have the ability to learn about each individual rather than the human population as a whole. However, novel humans will present a challenge to such animals as they have no prior information upon which to base their response. As a result, the benefits of IR may only become apparent over time, or when the number of humans in the population is small (electronic supplementary material, figure S6a). IR is expected to only be advantageous when the human population is heterogeneous (i.e. there are both dangerous and non-dangerous humans; see electronic supplementary material, figure S6b for an example of IR in a homogeneous human population). Given that IR gives critters the flexibility to learn human traits individually, a high learning weight should always be beneficial as it serves to accelerate convergence on the optimal strategy in human populations of any composition (electronic supplementary material, figure S6b). This contrasts with generalized learning, where human traits can only be learned at the population level and high learning weights can generate negative effects because of learning asymmetry (see §3.2).

### 4.2. In-between all or nothing: varying the degree of discrimination

As animals are unlikely to completely generalize or completely discriminate among individual humans, it is important to understand the effects of incomplete discriminative abilities; that is, when *D* is greater than 0 but less than 1. Certain cues are possessed by almost all humans (for example, two forward-facing eyes, upright posture, bipedalism), and these are likely to be particularly salient. By contrast, those human traits facilitating IR (such as face shape, hair colour and walking gait) may be less so. Our IR parameter thus approximates this trade-off between responding to shared versus individual cues.

**Figure 3.**
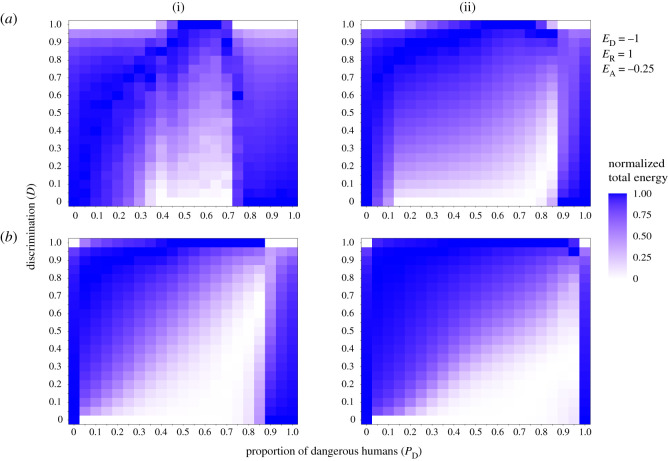
Heat maps demonstrating how changing the proportion of dangerous humans in the population affects the utility of varying degrees of discrimination (*D*) in terms of total energy gained or lost over time. In these scenarios, only populations with dangerous and rewarding humans are considered; neutral humans are ignored as they cause no change in energy. Thus, a value of 0 dangerous humans indicates a scenario where all humans are rewarding. Dangerous humans cause a decrease in energy equal to the gain in energy caused by rewarding humans (i.e. −1 versus +1) and the cost of avoiding is 0.25. Because the proportion of dangerous humans in the population determines the absolute possible final energy value, the energy values have been normalized to be between 0 and 1 within each level of human population composition. Values are therefore relative within columns along the *x*-axis: white squares indicate the worst performing *D* value for a given proportion of dangerous humans and shaded squares indicate how much better alternative levels of *D* fare in a given scenario. Row (*a*) shows a low learning weight of 0.1 and row (*b*) shows a high learning weight of 0.9. Column (i) shows energy after 500 encounters and column (ii) shows energy after 3000 encounters. *N*_humans_ = 100; *N*_critters_ = 500.

Generalized learning (*D* = 0) outperforms full IR (*D* = 1) in human populations with low heterogeneity (figure 3, shown by dark blue shading). As the heterogeneity of the human population increases, full IR, which facilitates only the avoidance of known dangerous humans, becomes the better strategy. Additionally, as the number of encounters increases (figure 3, column ii versus i), there is more time for the benefits of IR to accumulate, and IR becomes a more viable strategy over a greater range of human population compositions. Even a modest ability to discriminate among humans can result in relatively high energy gains and outperform both full IR and generalized learning in some scenarios. However, when the learning weight is high (figure 3*b*), learning asymmetry causes critters with any degree of generalization to avoid both familiar and unfamiliar humans, which causes those in mostly dangerous but heterogeneous populations to fare worse than conspecifics with full IR (*D* = 1). These simulations provide an indication of when animals may be able to benefit from using a combination of discrimination and generalization, and the precise scenarios where full IR or complete generalization are favoured. Note that substituting rewarding for neutral humans generates qualitatively similar results (electronic supplementary material, figure S7).

## 5. Social learning

Many animals learn via SL, which is defined as ‘learning that is facilitated by observation of, or interaction with, another individual or its products' [25]. SL may occur as a result of observational conditioning, whereby an observer forms a relationship between a stimulus (such as a human) and a demonstrator's response [25,26]; the observation of conspecifics is likely to be important in learning about humans. Some species (American crows, *Corvus brachyrhynchos* [17]; Eurasian jackdaws, *Corvus monedula* [27]) appear to learn the characteristics of individual humans in this manner and scold or mob dangerous humans more frequently than non-dangerous humans. SL can also take the form of social facilitation, whereby the mere presence of other individuals can alter an animal's behaviour [25]. Social facilitation can result in wild animals becoming habituated to humans after being attracted to foraging grounds by conspecifics [28]. We therefore also included SL in the model. With SL, critters can observe one conspecific from the critter population at each time step as well as encountering a human themselves. Thus, the learning equation for SL is identical to the equation for asocial learning (equation (3.3)), though the degree to which critters learn by SL is scaled by the social learning coefficient, *S*, which takes a value between 0 and 1. When *S* = 1, critters learn as much from conspecifics as they would if they encountered the human themselves, but do not experience a change in energy as a result. For a fuller exploration of the effect of changing *S*, see electronic supplementary material, figure S8. Here, we first consider a situation where critters do not learn from conspecifics that avoid encounters with humans and its effect at the critter population level when there is a change in the human population composition. We then consider a situation where critters with IR use alarm signals to spread information obtained from conspecific avoidance to others in the critter population.

### 5.1. Social learning can reverse suboptimal avoidance in a changing environment

Critters with SL are able to observe conspecifics, which means they can observe others' encounters with humans even if they have learned to avoid humans themselves. This has the potential to allow critters to overcome the problem of learning asymmetry generated by asocial learning (§3), which may have implications for when human populations change in composition over time. Examples of such change include hunting seasons and tourist seasons, when numbers of dangerous and rewarding humans in the population may temporarily increase, respectively. Animals that have learned to avoid humans in the hunting season could be disadvantaged when the human population becomes less dangerous. It would thus be beneficial for animals to be able to alter their behaviour to match the optimal strategy in changing environments. There is evidence that wild animals do adjust their responses to human activity; for instance, mouflon (*Ovis gmelini musimon*) show greater avoidance of humans in the hunting season than in the tourist season [29]. We therefore further extended the model to allow a temporal shift in the human population composition, to assess the effect of SL on suboptimal avoidance behaviour in critters that generalize (*D* = 0).

**Figure 4.**
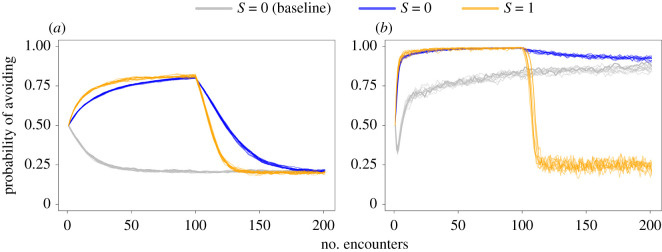
The effect of social learning (SL) on critters' ability to adjust their avoidance behaviour following a change in human population composition. After 100 encounters, the human population is reduced from 80% dangerous to 20% dangerous. Blue lines depict critters without SL (*S* = 0), orange lines depict critters with SL (*S* = 1), and grey lines indicate the baseline probability of avoiding without SL when the human population is composed of 20% dangerous humans at the beginning of and throughout the time series. Results are shown for critters with a low learning weight of 0.1 (*a*) and a high learning weight of 0.9, showing the associated effect of learning asymmetry (*b*). Figure shows 10 repeated simulation runs.

Specifically, we looked at the ability of critters with (*S* = 1) and without SL (*S* = 0) to modify their avoidance behaviour when the human population becomes less dangerous. First, we ran a simulation with a population of 80% dangerous humans for 200 time steps, such that there was a high level of avoidance behaviour in the critter population. We then ran a simulation that included a change in the human population composition, reducing the number of dangerous humans to 20% after 100 time steps. At this point, the previously optimal strategy of avoiding is now suboptimal. Figure 4 shows the change in *P*_A_ over time for critters with (orange) and without (blue) SL in this scenario. When the learning weight is low (*W* = 0.1, figure 4*a*), critters can converge on the same *P*_A_ as they would if they had not previously experienced a different human population composition. SL speeds up convergence on this strategy but does not alter it greatly. By contrast, when the learning weight is high (*W* = 0.9, figure 4*b*), the *P*_A_ of critters without SL barely changes from 1 (always avoid) because of learning asymmetry (see §3.2), while those with SL quickly converge on the same strategy as critters with the lower learning weight. SL thus provides critters that have already learned to avoid humans with an opportunity to discover a more optimal strategy.

### 5.2. Alarm signals upon avoidance improve the effectiveness of individual recognition

**Figure 5.**
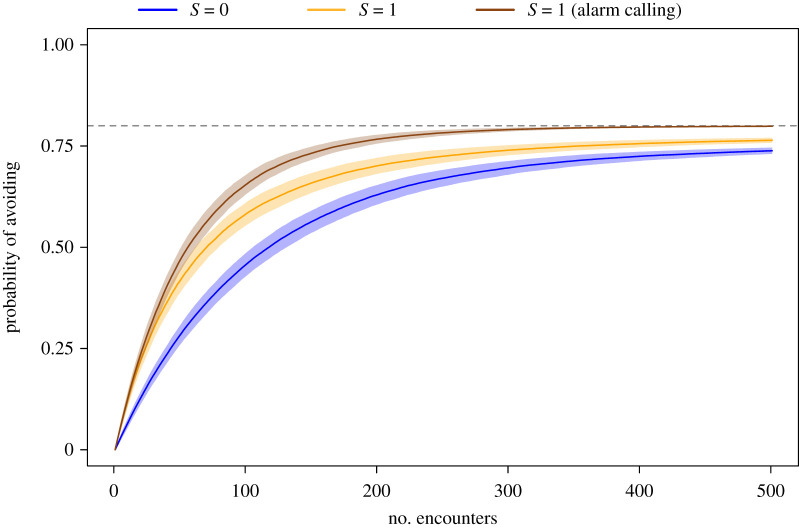
Time series showing the effect of SL on the ability of critters with IR (*D* = 1) and a high learning weight (*W* = 0.9) to reach the optimal avoidance strategy in a population of 80% dangerous humans. Here, three scenarios are shown: one where critters do not socially learn (*S* = 0; blue line), one where critters socially learn from observing the nature of conspecific interactions alone (*S* = 1, no alarm signalling; orange line) and one where critters also learn from conspecifics' avoidance of a dangerous human (*S* = 1, alarm signalling; dark brown line). Lines show critter population mean ± s.e. probability of avoiding at each time step. The optimal avoidance strategy is indicated by a dotted line.

We have considered scenarios where critters do not learn from another's avoidance. However, many species produce alarm signals upon fleeing a perceived threat [30], which function to alert others to danger [26]. Furthermore, alarm signals such as calling and mobbing have been shown to facilitate IR of dangerous humans [17]. Some animals can even learn to recognize individual humans based on their pairing with negatively valenced vocalizations alone: for example, jackdaws (*C. monedula*) are more likely to scold humans that previously have been paired with playbacks of scolding calls [27]. As a final exploration of how SL affects animals' responses to humans, we modelled these alarm signals by allowing critters with IR to also learn from conspecifics that avoid humans. It may be expected that an animal that is capable of recognizing individual humans would only produce alarm signals when a human is known to be dangerous. We therefore set the initial *P*_A_ to 0, as this enables alarm signalling only for dangerous humans rather than any newly encountered human (starting with a *P*_A_ of 0.5 would introduce misinformation into the critter population). Critters can then learn from conspecifics' later avoidance of these individuals, allowing the information spread through the critter population. Indeed, we find that the inclusion of alarm signals enables critters to reach their optimal strategy far sooner than when critters do not use such signals (figure 5). This highlights the potential role of SL in making IR a more viable strategy, and the importance of good discriminative abilities for spreading correct information about the level of threat each human poses. See electronic supplementary material, figure S9 for an exploration of the effect of alarm signalling on the avoidance behaviour of critters that generalize (*D* = 0).

## 6. Discussion

In many parts of the world, wild animals live alongside humans. The challenges that arise from their encounters have a profound effect on behaviour and, ultimately, survival. In particular, humans differ greatly in how they behave towards animals, and animals potentially differ in the strategies they use to cope with human disturbance, both of which will influence whether encountering humans is beneficial, inconsequential or detrimental. Using our simulated population of ‘critters’, we examined how animals can converge on an optimal avoidance strategy in environments where human behaviour may vary. Specifically, we considered how cognitive processes such as a fast learning rate, IR and SL affect critters' avoidance strategies when encountering humans, and ultimately their energetic outcomes.

### 6.1. How to learn about humans if they all appear to be the same?

When critters generalize from their encounters with humans, they are unable to discriminate among individuals and all humans are treated as the same type of stimulus. This may be an accurate representation of humans for many animal species: consider how it is often impossible for humans to differentiate among members of another species unless they are purposefully marked or tagged. Furthermore, cues that appear salient to humans might not necessarily appear salient to other animals [31]. If there is any uncertainty regarding the discriminability of the cue, it may be far more beneficial to flee than to remain and risk being injured or killed [32]. We showed that generalization is a good strategy when human populations are homogeneous in their behaviour. However, the constraint imposed by generalizing experiences to the whole human population makes it very difficult to reach an optimal strategy when there is a moderate amount of heterogeneity in human population composition and humans thus give ‘mixed messages’.

### 6.2. Learning rate and the potential for a perceptual trap

Learning rate is determined by how much weight animals place on their most recent experiences compared with their previous experiences [33]. A higher learning weight results in a faster rate of learning at the cost of potentially discarding information from older experiences. As such, a fast rate of learning may only be useful when the stimulus, in this case, humans as a class, is consistent; otherwise, animals would be sensitive to random fluctuations in their environment that might not be truly informative. With our model, we showed that, when the learning weight is increased, critters become more likely to avoid humans if there are any dangerous humans in the population. This is because a high weighting of new encounters reveals an asymmetrical effect: a high learning weight means animals are more likely to avoid humans in the future after experiencing a threatening encounter, and, once animals have learned to completely avoid a stimulus, they cannot learn to stay. A fast learning rate can therefore be beneficial or detrimental depending on how dangerous the human environment is. A high learning weight generates high avoidance, but, if the population is not sufficiently dangerous to warrant such behaviour, it essentially creates a perceptual trap [34].

However, there may be times when even a small number of humans (for example, hunters) present a high level of threat and it would be costly to risk staying, so high avoidance after even a single encounter could be beneficial. At a low learning weight, learning tends to be more symmetrical, with critters staying frequently enough to learn from rewarding and neutral encounters as well as dangerous encounters. Therefore, when there is an equal number of dangerous and non-dangerous humans in the population, critters' probability of avoiding remains close to chance, with critters tending to avoid for the average proportion of encounters they have with dangerous humans. As there is no ability to discriminate among humans, their avoidance behaviour is independent of the occasions when they actually encounter dangerous humans, and they are unable to reach an optimal avoidance strategy.

It is worth considering whether the learning asymmetry we demonstrate in our model would generalize to other modelling frameworks. Any mechanism that reduces random fluctuations in the type of human encountered or allows critters to learn about humans despite having opted to avoid them is expected to disrupt this process. Other learning models (such as [35,36]) emphasize the trade-off between exploration of possible options and exploitation of accumulated knowledge, where exploration increases the randomness of the decision-making process. If such additional stochasticity was added to the decision-making process in our model, we expect it would also disrupt the trap of avoidance by allowing critters to occasionally stay and therefore learn. Hence, high exploration rates would mitigate learning asymmetry but could limit critters' ability to approach optimal avoidance strategies.

### 6.3. Recognizing individual humans

IR of humans enables animals to escape the problems generated by the mixed messages of heterogeneous human populations. When animals have full IR, they are able to exhibit a discrete response to each human, fleeing only from dangerous individuals. This means that a high learning weight is always better, providing that individual humans are consistent in their behaviour and animals have identified individuals correctly. The benefits of IR are greater and manifest earlier when the human population is heterogeneous, in particular, when the number of dangerous humans in the population is intermediate. Animals that fully discriminate among individual humans and live amid homogeneous human populations will fare poorly early on compared with animals that learn predominantly through generalization. Because of this, lifespan could be important: the benefits of human IR often only become apparent over time and, as such, only longer living species may be able to make use of it. Animals that recognize individual humans must have the capacity to remember them over a period of time; indeed, our model was not designed to consider memory constraints. How many individuals an animal can remember, and for how long, is a critical question and likely to be important for understanding which species are capable of IR and how human IR can develop. Most of the species that have exhibited human IR have been corvids [23,27,37–39], a family of birds known for their relatively large brain size, complex cognition and long lifespans [40], although it is not clear whether there has been a bias towards testing corvids for this ability. Nevertheless, storing and accurately remembering the cues associated with multiple humans over extended periods of time is likely to be cognitively demanding and may only be expected in species that have already evolved the ability to recognize individual conspecifics [41].

### 6.4. Beyond ‘true’ individual recognition

Relatively few studies show the ability of free-living, wild animals to recognize individual humans or other conspecifics, and, to our knowledge, none demonstrates what would be considered ‘true’ IR; that is, when cues are associated specifically with one individual and no other [42]. This, however, may simply be because experiments investigating IR in wild animals have not been designed to explicitly test this hypothesis, instead measuring behavioural responses to a relatively small number of human stimuli (either individuals or masks). It is therefore unknown whether the bird species (e.g. mockingbirds [15], brown skuas [24]) that have demonstrated IR have truly recognized an individual rather than responded to a particular cue that could either be shared by another individual (for example, the same hairstyle) or isolated (i.e. animals would react in the same way if the relevant cue were somehow abscised and presented alone). While assessing the specificity of individual human cues was outside the scope of our study, we did approximate a situation where there is neither complete discrimination nor complete generalization of human individuals. We showed that an intermediate level of discrimination, whereby critters learn strongly about a particular individual as well as generalizing about all humans in the population to a lesser extent, can result in similar outcomes to pure discrimination or generalization, often achieving close to optimal avoidance behaviour across a wide range of scenarios. As animals will not be able to predict the human environment they find themselves in, nor whether the next encounter will be dangerous or benign, it appears to be useful to balance generalization and discrimination. This finding may explain why, in the studies of IR of dangerous humans in birds, subjects often display at least some defensive behaviour to behaviourally neutral human stimuli [15,23]. Thus, it appears unlikely that these species display true IR, although it of course cannot be ruled out that perception and responses are not necessarily always coupled. For example, animals may regard an unfamiliar or less familiar human with wariness until they have gained sufficient experience of them. Furthermore, it may be rare for animals to encounter the same individual repeatedly. It is therefore possible that some wild animals may be able to recognize individual humans but do not behave as such because doing so might be costly. In some situations, classifying humans at a group level, rather than discriminating among them individually, could be more beneficial. This may be the case for African elephants (*Loxodonta africana*) in Kenya's Amboseli National Park, where it is predominantly Maasai men who hunt elephants [43].

### 6.5. Social learning about humans

Many animals are capable of SL and a small number of studies demonstrate the ability of animals to socially learn the characteristics of dangerous humans [17,27]. To our knowledge, no studies have shown SL of rewarding humans, although there is good reason to assume that it could occur: observational conditioning of rewarding stimuli has been documented in several species, such as feral pigeons [44] and bumblebees (*Bombus terrestris*) [45]. We found that SL can reduce the number of encounters with humans required to reach the equilibrium avoidance strategy. Perhaps more importantly, our model suggests that SL could help animals overcome the problem of learning asymmetry, as, through observing conspecifics foraging alongside humans without harm, they will become less likely to flee. This will encourage wary animals to learn to tolerate humans and may enable conspecifics that have learned to avoid humans through asocial learning to become less wary in the future, should the level of threat decrease.

Animals that socially learn the characteristics of dangerous individual humans may use alarm signals, which facilitate the spread of information through a population [17]. Exclusively producing negatively valenced signals when an individual is a known threat is a useful way of learning about the human population more quickly and without having to face the risk of a direct encounter, thus reducing costs. At least two species that have been tested for human IR have been later assessed for SL of individual humans [17,27], suggesting that SL may be an important ability to function alongside IR. Our model supports this: we found that SL increases the utility of IR by enabling the optimal avoidance strategy to be reached sooner. Additionally, it is plausible that animals may learn more from SL than from asocial learning: while animals cannot learn if they are killed, the death of conspecifics provides useful information [46].

In our model, individuals can learn directly by observing conspecifics. Our SL parameter, *S*, controls how salient observed encounters are (with *S* = 1 being as salient for the critters as experiencing the encounter themselves). It is common to model SL as a trade-off between using individual experience and social cues when making a decision (e.g. [35,36]). Therefore, social cues affect individuals' decisions, but individuals only learn through direct experience. We do not expect there to be major differences between the two approaches in the average case (i.e. after many observed encounters), but since critters can learn directly from observing conspecifics, our approach lends itself to modelling socially learned avoidance.

### 6.6. Humans as a discrete stimulus

Our critters were simulated in an environment free from other predators. In reality, animals face predation and competition from other species besides humans, and therefore their behaviour in response to humans may be affected by these other encounters [47]. Animals may either be more or less likely to flee depending on the presence of other species in the landscape. This is most evident from studies of island tameness (e.g. [5]), which provide evidence that animals that have evolved alongside terrestrial predators show greater fear responses to humans, while those that evolved in the absence of predation pressure can be approached at close range and are thus most vulnerable to human activity. We expect that, if animals are not able to reasonably distinguish between humans and other large predators, and these other predators are present, avoidance of humans will be greater [13]. This may explain why some species that frequently encounter humans, but which are not at risk of predation by them, maintain wariness of humans.

There is some evidence, however, that wild animals do distinguish between humans and other predators. In some scenarios, ‘prey’ species such as deer may choose to stay close to areas with high human disturbance, as these areas are less commonly used by large carnivores [48]. In other scenarios, the threat posed by human populations may exceed the threat posed by these carnivores, causing greater avoidance of humans than of other predators [49]. The relative risk posed by other predators is therefore an important factor in understanding wild animals' responses to humans.

### 6.7. How do animals respond to humans in real life?

Our model is simple: we forced our agents to make a dichotomous choice between staying and avoiding. In reality, although animals flee from humans (a behaviour exploited by flight initiation distance experiments such as [50–52]), they may also use intermediate strategies such as maintaining a certain distance, and be unable to escape injury because of humans' use of projectile weapons. Wild animals may also change their spatial distribution according to the location of human populations that differ in their level of threat or to the habitat use of other predators [48]. In any case, our model approximates this decision-making behaviour.

We assume that dangerous encounters cause a loss in energy and create the ability for animals to learn concordantly from them. As animals cannot learn from their encounters if they are killed by them, we did not include death in our agent population, and thus we cannot model how lethal encounters affect conspecifics' responses to humans. It is possible that dangerous encounters that are not injurious may not be particularly costly. For example, if the human's objective is to deter animals from a location, they may only be harmed if they do not flee. Even if the risk of death at any one time is very low, animals may need to maintain a high level of wariness to ensure that they are able to avoid being killed or injured over a longer time period. Although animals tend to learn faster about dangerous stimuli than rewarding stimuli [53], it may be possible that animals are able to evade harm in many cases and thus learn more strongly from rewarding encounters with humans. Indeed, animals will habituate to an aversive stimulus if it benefits them to ignore it [54]. Maintaining an appropriate level of wariness may be difficult if animals learn to approach humans as a result of being fed, and could leave them vulnerable to negative encounters [10].

On the other hand, animals would benefit from choosing an option that always minimizes the risk of death or severe injury and therefore may behave in such a way that yields lower total energy from foraging if the predation risk is sufficiently high [55]. Moreover, there are diminishing returns in the survival benefits associated with increasing energy intake, because animals need only consume a certain amount of food to survive. A well-fed animal might therefore be more averse to predation risk by humans. These factors are likely to affect foraging decisions in the presence of humans. Our model considers a situation where animals are under pressure to meet their daily caloric intake, and the advantage gained from increasing energy is constant, but this will not always be the case in wild systems.

### 6.8. The implications of feeding wild animals

While there has been ample research on the negative effects of humans on wild animals and many accounts of humans feeding wildlife [10], relatively little research has been conducted on the effect of direct feeding interactions on wild animal behaviour. However, there is evidence that food provisioning can alter large-scale behaviour patterns, such as the migratory behaviour of birds [56]. Additionally, anthropogenic food may improve the body condition of animals that consume it [57], but food provisioning may have detrimental effects in the longer term, for example by encouraging the abandonment of other beneficial behaviours or by increasing disease transmission risk [58]. Unintentional food provisioning that encourages animals to approach humans and damage anthropogenic resources, such as crops, can result in humans resorting to lethal control to protect themselves and their livelihoods [59,60]. The implications of widespread food provisioning by humans are potentially important for avoiding or mitigating conflict, particularly when interactions with wild animals are unwanted or pose a threat to species of conservation concern.

## 7. Conclusion

Knowing how to behave in the presence of humans may be one of the most difficult challenges wild animals face. As individual humans may act very differently from one another towards wild animals, they thus send ‘mixed messages’, to which animals must respond effectively in order to succeed in human-dominated environments. There are a few ways by which animals can do this, but their effectiveness depends on the relative number of dangerous humans present, the frequency of encounters, and how quickly animals can learn. An inability to learn quickly may be an issue for species that have little experience of humans but which are subject to consumptive activities such as hunting, trafficking and persecution.

Conversely, animals that learn quickly are vulnerable to falling into a trap of avoidance despite there being a low likelihood of danger. The ability to learn socially can help animals overcome this perceptual trap, as they can gain information from observing others foraging alongside humans without harm; this could be especially effective when the human population composition fluctuates over time, for example, because of hunting or tourist seasons. SL can also accelerate convergence on a more optimal avoidance strategy and may be particularly valuable when animals are capable of recognizing individual humans. The ability and utility of IR is likely to require fast learning, although animals need not fully discriminate among humans to reap considerable benefits over counterparts without this ability.

Animals are unlikely to use or require full IR, but some ability to discriminate among humans will always be beneficial when different, but repeatedly encountered, humans engage in contrasting behaviours. Further research is required to reveal the specificity of the cues used to identify individuals, how sensitive animals are to them, how many individuals can be remembered, and the length of time for which individuals can be recognized, as well as whether wild animals are capable of what can be described as ‘true individual recognition’. Furthermore, whether animals view humans as a category separate from other species, and thus are able to respond to them independently, is still unknown. Fully deciphering the mechanisms underlying the recognition of humans will be valuable in predicting how animals respond and adapt to human activity, and in understanding which species may be most vulnerable to human exploitation.

## Data Availability

The model code has been provided. Additional figures are provided in the electronic supplementary material [61].
